# Spatiotemporal Distribution and Genetic Characterization of Measles Strains Circulating in Greece during the 2017–2018 Outbreak

**DOI:** 10.3390/v12101166

**Published:** 2020-10-15

**Authors:** Vasiliki Pogka, Elina Horefti, Maria Evangelidou, Evangelia Georgia Kostaki, Dimitrios Paraskevis, Anastasia Flountzi, Theano Georgakopoulou, Ioanna Magaziotou, Andreas Mentis, Timokratis Karamitros

**Affiliations:** 1 National Measles and Rubella Reference Laboratory, Department of Microbiology, Hellenic Pasteur Institute, 11521 Athens, Greece; vpoga@pasteur.gr (V.P.); horefti@pasteur.gr (E.H.); meuagelidou@pasteur.gr (M.E.); mentis@pasteur.gr (A.M.); 2 Department of Hygiene, Epidemiology and Medical Statistics, Medical School, National and Kapodistrian University of Athens, 15772 Athens, Greece; ekostakh@med.uoa.gr (E.G.K.); dparask@med.uoa.gr (D.P.); 3 European Program for Public Health Microbiology Training (EUPHEM), European Centre for Disease Prevention and Control (ECDC), 16973 Stockholm, Sweden; fanastasia@hotmail.com; 4 National Public Health Organization, 15123 Athens, Greece; t.georgakopoulou@eody.gov.gr (T.G.); i.magaziotou@eody.gov.gr (I.M.); 5 Bioinformatics and Applied Genomics Unit, Department of Microbiology, Hellenic Pasteur Institute, 11521 Athens, Greece

**Keywords:** measles, measles virus, epidemic, Greece, outbreak, spatiotemporal

## Abstract

Between May 2017 and November 2018, Greece has experienced a severe measles outbreak with a total of 3258 cases reported, after reaching its goal of eliminating measles since 2014–2015. In this study, we aimed to investigate the origin and the dispersal patterns of the measles strains that circulated in Greece during this outbreak and to identify possible transmission patterns of measles virus (MeV) in the country. Of the 832 measles suspect cases referred to the National Measles and Rubella Reference Laboratory for MeV RNA detection, 131 randomly selected positive samples, representative of the temporal and spatial distribution of the laboratory-confirmed measles cases in Greece, were processed for genotypic identification by an RT-PCR amplification of a 598 bp fragment containing the 450 bp hypervariable region of the measles virus N gene. Phylogenetic analysis was carried out by the approximate maximum likelihood method (ML) under the generalized time-reversible (GTR + cat) model. All samples analyzed were found to belong to genotype B3. Comparative analysis with other European and reference measles strains revealed three separate major clusters and other multiple viruses circulating simultaneously in Greece. They were all isolated from three main community groups, Greek-Roma children, non-minority Greek nationals and immigrants/refugees, a finding that is in accordance with what was also observed in the last two measles outbreaks in 2005–2006 and 2010–2011. Notably, for one of the three clusters, no similarity was detected with previously reported prototype strains. Our results indicate the need for a more intensive vaccination program against measles amongst minority populations and in refugee hot-spots as well as the importance of molecular surveillance as a tool for monitoring measles outbreaks.

## 1. Introduction

Measles Virus (MeV) belongs to the family Paramyxoviridae and to the genus Morbillivirus. It is an enveloped virus with a negative-sense, single-stranded, 15.6 Kb-long RNA genome. MeV is classified into eight clades, A-H, and 24 genotypes, five of which are inactive [1]. Measles is an acute viral illness, with symptoms like fever as high as 40 °C (105 °F), malaise, cough, coryza, conjunctivitis and a characteristic maculopapular rash, which usually appears 14 days after the initial exposure to the virus. The illness is generally characterized as mild or moderately severe. However, sometimes measles may result in complications such as pneumonia, encephalitis and even death, with the post-infectious encephalitis occurring in approximately one in 1000 reported measles cases. Finally, for every 1000 reported measles cases, almost two deaths are likely to occur [2].

According to European Vaccine Action Plan (EVAP), all Member States have endorsed the strategy of eliminating measles by 2020 in the European Region [3]. Measles elimination is defined as the absence of endemic measles virus transmission in a region or other defined geographic area for ≥12 months. National surveillance systems are implemented by all Member States in order to detect all clinical cases of measles as well as to investigate all cases and outbreaks [4]. 

Although a safe and cost-effective vaccine has been available for decades, measles is still an ongoing public health problem in the European Region. At the beginning of the 21st century, 37,421 measles cases were reported in the European region; their number declined progressively to 4363 cases reported in 2016. Various outbreaks in the region resulted in 24,356 and 89,148 cases in 2017 and 2018, respectively [5]. In Greece, between 1990 and 2000, 12,250 measles cases were reported; since 2000, two outbreaks occurred in the country, in 2005–2006 and in 2010–2011, with 636 and 189 laboratory-confirmed cases, respectively. Phylogenetic analyses showed that the co-circulation of D4 and D6 genotypes was responsible for the 2005–2006 outbreak [6], whereas the 2010–2011 outbreak was due to the D4 genotype [7]. In both outbreaks, the social groups mainly affected were Roma–Greek children and young adults of Greek citizenship. 

Greece had successfully prevented measles since 2014–2015 [8,9]. However, a measles outbreak started in May 2017, with a total of 3258 cases up until November 2018, according to the National Public Health Organization (NPHO) [10,11]. The purpose of this study is to identify the genetic identity of the measles strains that circulated in Greece during this outbreak, as well as to present their spatial and geographical distribution, in order to identify the sources of importation and transmission patterns of the virus in the country.

## 2. Materials and Methods

### 2.1. MeV RNA Detection

Of the 832 measles suspect cases referred to the National Measles and Rubella Reference Laboratory for measles RNA detection (809 pharyngeal swabs, 18 plasma, 3 whole blood and 2 cerebrospinal fluid), 713 (85.7%) were confirmed as positive by real-time PCR. Viral RNA was extracted from 200 μL of clinical sample using the NucliSens easyMAG automated system (BioMérieux, Marcy l’Etoile, France). After cDNA synthesis using SuperScript III reverse transcriptase (Invitrogen), a 114 bp fragment of the N gene was amplified according to Hübschen JM et al. [12].

### 2.2. MeV Genotyping

One hundred thirty-one randomly selected, fully anonymized, MeV RNA-positive samples (Table 1), representative of the temporal and spatial distribution of the laboratory-confirmed measles cases in Greece (targeting approximately 20% of all positive samples from each province), were retrospectively processed for genotypic identification by a RT-PCR amplification of a 598 bp fragment containing the 450nt-long hypervariable region of the measles virus N gene, encoding the 150 carboxy-terminal amino acids of the N protein (N450) [13]. Each amplicon was bidirectionally sequenced to resolve the possible presence of ambiguous nucleotides. Sanger Sequencing was performed using the BigDye ™ Direct Cycle Sequencing Kit (Thermo Fisher Scientific, Waltham, MA, USA) on the ABI3730xl DNA Analyzer (Thermo Fisher Scientific, Waltham, MA, USA).

### 2.3. Phylogenetic Analysis

In order to further study the relationships between the Greek strains and those circulating in other countries, the analysis was supplemented with additional homologous sequences available from the measles nucleotide surveillance (MeaNS) database [14], using BLAST to compare all unique sequences from our dataset to the MeaNS database and keeping 10 sequences each time, those presenting the higher BLAST score. After comparison of all BLAST results, duplicated sequences were removed, and their availability in GenBank was also confirmed. Phylogenetic analysis was carried out by the approximate maximum likelihood method (ML) under the generalized time-reversible (GTR + cat) model of nucleotide substitution model including a Γ-distributed rate of heterogeneity among sites, as implemented in FastTree version 2.1 program [15]. Phylogenetic clusters were defined using two different criteria: (i) clusters with Shimodaira–Hasegawa (SH) values greater than 0.95 or clusters consisting of identical sequences (phylogenetic confidence criterion) and (ii) clusters consisting of at least 5 sequences sampled in Greece at a proportion greater than 80% compared to the total number of sequences within the cluster. All sequences obtained in this study were submitted to MeaNS with restricted users’ access permission.

## 3. Results

**Figure 1:**
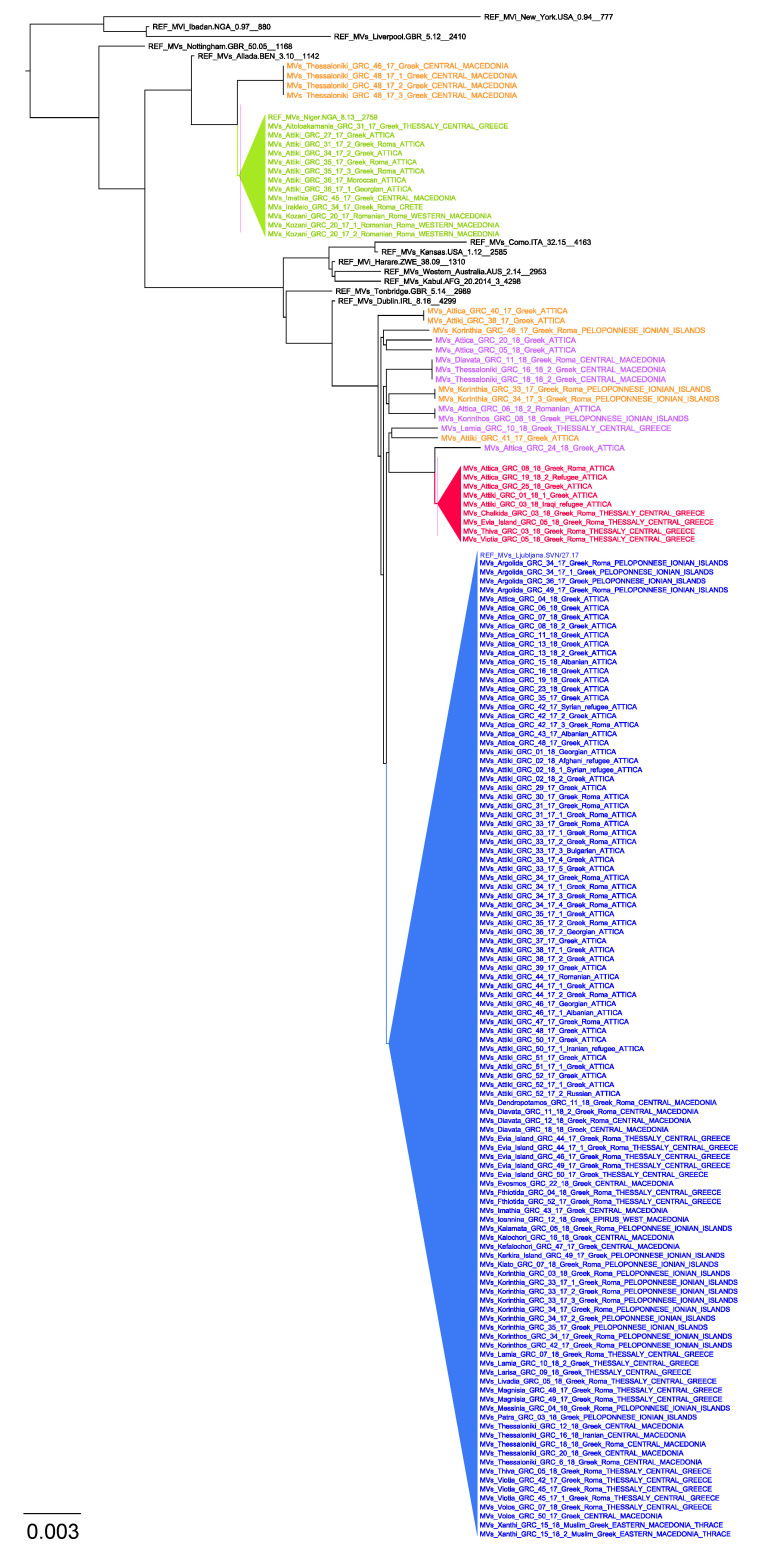
Evolutionary relationships of measles viruses circulating in Greece with other European and named strains from measles nucleotide surveillance (MeaNS) database and WHO reference strains for genotype B3, based on the 450 nucleotides sequence encoding the 150 carboxy-terminal amino acids of the N protein (N450). Three main circulating clusters are indicated in green, red and blue triangles. Lineages sampled in 2017 and 2018 and cocirculated with the main clusters are colored in orange and purple, respectively.

Seven hundred thirteen (85.7%) samples out of the 832 measles suspect cases referred to our laboratory for measles RNA detection were confirmed as positive by real-time PCR (695 pharyngeal swabs, 15 plasma, 2 whole blood and 1 cerebrospinal fluid). Sequence analysis was performed in 131 positive measles cases and indicated that they all belonged to genotype B3. Further phylogenetic analysis including other European and reference measles strains revealed three separate clusters and multiple viruses circulating simultaneously in Greece (Figure 1). 

**Figure 2:**
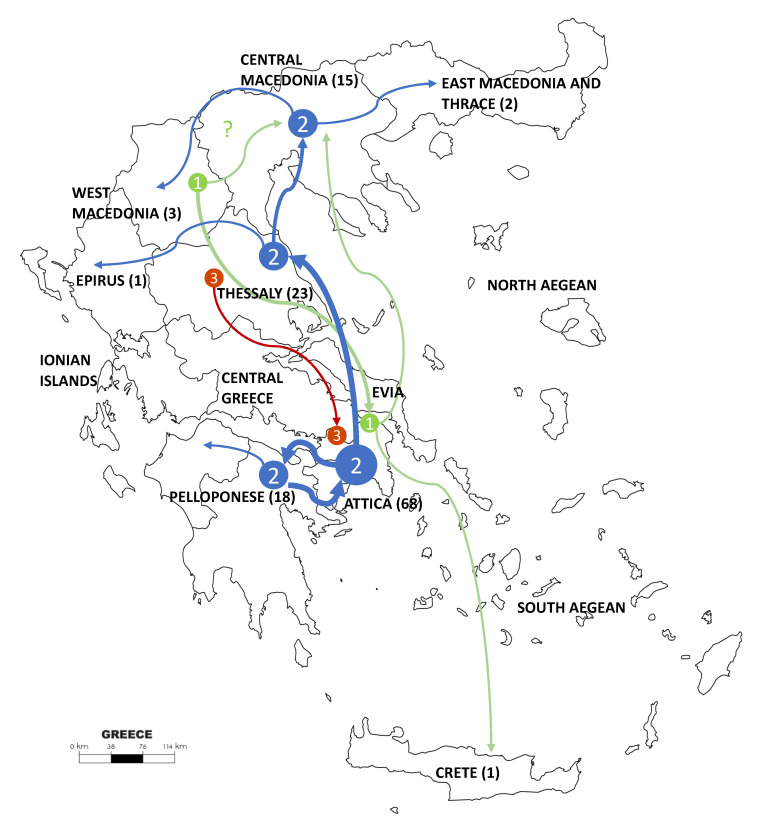
Geographic representation of the three main measles virus (MeV) clusters’ dispersal across Greece during the 2017–2018 outbreak. Colored arrows and numbers are in accordance with Figure 1 and Figure 3A. The flow is based on locations of samples isolation across time. The number of sampled sequences from each province is presented in parentheses.

**Figure 3:**
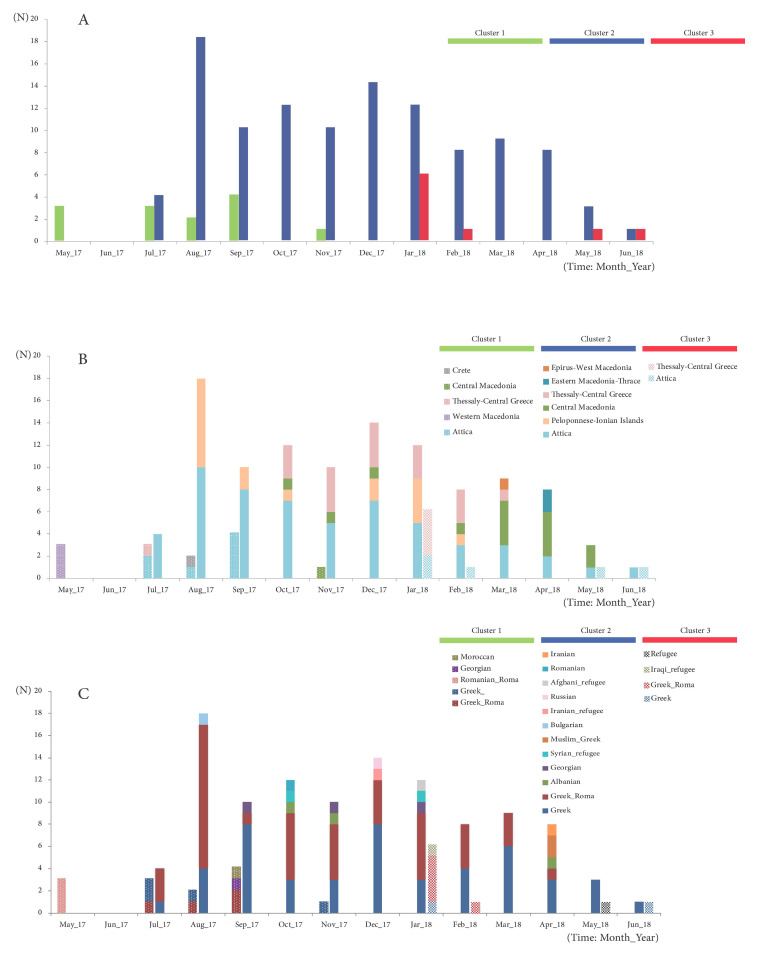
Epidemic curves of (**A**) the 3 main circulating clusters of the 2017–2018 MeV epidemic in Greece, broken down according to (**B**) origin (administrative regions of Greece) and (**C**) demographic characteristics of the infected individuals.

Numbered in order of appearance, Cluster 1 was initially dispersed from Northwest to South and included 13 viral sequences, which were 100% identical to the MVs/Niger.NGA/8.13 reference strain (Figure 1 and Figure 2). Cluster 1 was the dominant B3 sequence variant at the beginning of the outbreak (Figure 3A) and circulated mostly in Northern and Central Greece, as well as in Attica and in Crete (Figure 3B). Cluster 2 was the largest, detected mainly in Attica and Peloponnese-Korinth before its dispersal to the North and the rest of the country. It was first isolated in July 2017 and continued to circulate until the end of the outbreak, June 2018, including 109 viral sequences that were identical to the WHO named strain MVs/Ljubljana.SVN/27.17 (Figure 1). Cluster 3 included nine viral sequences, which circulated only in Thessaly and Attica and it was the smallest variant group detected in Greece (Figure 3B). This cluster, which showed no high similarity with any of the known WHO reference strains, appeared last, in January 2018, and lasted until the end of the outbreak as well. Apart from the three main epidemic clusters, multiple variants were detected throughout the course of the epidemic and across all affected regions.

Positive measles cases belonged to three major community groups: Greek–Roma children, non-minority Greek individuals and immigrants/refugees. The first confirmed cases of the outbreak that were isolated in May 2017 concerned three Roma siblings of Romanian nationality (Figure 3C), while the rest of the MVs/Niger.NGA/8.13 variants belonged to four Greek people, four Greek–Roma children and two foreigners of Moroccan and Georgian nationality. At the end of July 2017, the first cases of the MVs/Ljubljana.SVN/27.17 variant in a Greek individual and two Greek–Roma children were identified. Until June 2018, the vast majority of these strains involved Greek–Roma children, with 56 strains isolated from Roma camps in Central, Southern and Northern Greece. The same variant was isolated in 25 non-Roma children of Greek nationality, in six children from refugee camps originating from Iran, Iraq, Syria and Afghanistan and one adult from Iran and, finally, in 12 immigrant children who were born and raised in Greece. The remaining 26 strains belonged to Greek adults, whereas there were nine strains isolated from seven Greek individuals and two Greek–Roma of unknown age (Figure 3C). Finally, Cluster 3 variants involved two Greek individuals, five Greek–Roma children and two refugee individuals and co-circulated with the MVs/Ljubljana.SVN/27.17 variants from January 2018 until the end of the outbreak. Cluster 3 sequences, however, were detected at lower numbers in our study population than Cluster 2.

Regarding the geographical spread of the viruses belonging to the three different clusters, those from the first and second expanded more widely. Specifically, Cluster 2 was detected across Athens metropolitan area and a neighboring province in Peloponnese and all over continental Greece. Viruses classified within Cluster 1 were spread from Central Greece to Crete and to the North and Northwest. For Cluster 3, more limited spread was detected, including only two areas in Attica and Thessaly. The most putative epicenter at the early stage of the epidemic was in West Macedonia (cluster 1), while for the Clusters 2 and 3, which appeared later, the putative origin was Attica.

## 4. Discussion

According to the NPHO, from May 2017 to December 2018, a total of 3258 measles cases were reported in Greece, making this outbreak the largest one experienced so far in the country in the 21st century. Among them, 1162 were epidemiologically confirmed, 211 were classified as measles-positive based on their clinical symptoms and 1885 were laboratory confirmed. Sequence and phylogenetic analysis of 131 randomly selected positive samples indicated that they belong to genotype B3, which was in accordance with the recent outbreaks and epidemics of the virus in other European countries [16,17,18].

The first three laboratory-confirmed measles cases were reported in May 2017 and belonged to three Romanian–Roma siblings who seemed to have contracted the virus upon a trip to Romania. The viral strains detected in the first cases were identical to the MVs/Niger.NGA/8.13 variant of the B3 genotype, which was circulating in Romania during the same period [19,20]. Simultaneously to its progression, the MVs/Ljubljana.SVN/27.17 variant became dominant, and until November 2017, the two sequence variants co-circulated in the country. This is in concordance with previous findings from 28 European countries, which, as in 2017, reported several variants of the B3 genotype, with 23 reporting MVs/Dublin.IRL/8.16 as the dominant strain, corresponding to 70% of all B3 variants; the MVs/Niger.NGA/8.13 variant was detected at a lower frequency [21].

The presence of two major clusters indicates that the founder strains of measles transmission in Greece have been originated from different sources. As the MVs/Niger.NGA/8.13 variant was probably introduced from Romania, the putative origin of the MVs/Ljubljana.SVN/27.17 variant remains unknown, as it was isolated simultaneously in various parts of Greece and within different social groups. Moreover, there was no evidence of travel abroad among the early detected cases infected with MVs/Ljubljana.SVN/27.17 variant. According to a personal communication between the Greek and Slovenian National Reference Laboratories, the MVs/Ljubljana.SVN/27.17 strain probably originated from a one-year-old Syrian refugee infant who traveled from the Moria Greek refugee hotspot to Slovenia in July 2017. The child showed signs of measles infection while being in Serbia and was diagnosed with measles in Slovenia, where the subsequent genotyping took place. The same strain was also isolated in other European countries at the same time, like in Italy [22] and in Russia [23].

The third cluster showed no similarity to previously identified strains and was detected first among a Greek individual, Greek Roma and refugees in Attica and Thessaly. This cluster is of importance, showing that besides the prototype strains, additional viruses can seed the formation of outbreaks. Although the cluster did not expand across the country, it was isolated from at least two different locations. 

Furthermore, we found multiple circulating variants at low frequency, isolated across Greece. Given the relatively low substitution rate of the measles virus, these minority variants probably did not originate from the major clusters circulating in Greece, but rather, they represent multiple introductions from other European countries that contributed to the formation of a reservoir in the Greek population. The circulation of different variants indicates that different strains have been introduced in the population from different sources, thus increasing the risk of onward transmissions and ignition of outbreaks [24,25]. The establishment of transmission chains and the emergence of outbreaks are determined by various factors, including the epidemiological characteristics of the infected individuals and the levels of vaccination [26,27], but also the fitness of the viral variants themselves [28,29,30,31]. The existence of different seeds circulating at the same time increases the risk of onward transmissions and indicates the need for continuous molecular surveillance for early identification and monitor of outbreaks spread [32,33].

The epidemiological data of the 2017–2018 outbreak follow the same pattern with those of the 2005–2006 and 2010–2011 outbreaks. The social groups most affected were, in all three outbreaks, Greek–Roma children and Greek adults. This suggests the need for a more intensive vaccination program targeting more vulnerable populations such as Roma, refugees or other people, as well as the need for a second MMR-dose for adults. Finally, immunization against measles has to be intensified amongst populations in refugee hot-spots but also nationwide. The fact that in the 2017–2018 measles outbreak, children of Greek citizenship, as well as children of immigrants who were born and raised in Greece, were also affected shows gaps in the immunization of the general population, which have to be addressed.

## Figures and Tables

**Table 1:** Epidemiological characteristics of the genotyped MeV patients.

Total Number (N)	131
Average age (y)	11.06
Gender N (%)	
Male	62 (47.3%)
Female	69 (52.7%)
Province (N)	
Attica	68
Thessaly	23
Peloponnese	18
Central Macedonia	15
West Macedonia	3
East Macedonia and Thrace	2
Epirus	1
Crete	1
Nationality	
Afghani refugee	1
Albanian	3
Bulgarian	1
Georgian	4
Greek	57
Greek–Roma	50
Iranian	1
Iranian refugee	1
Iraqi refugee	1
Moroccan	1
Muslim Greek	2
Refugee Unknown	1
Romanian	2
Romanian Roma	3
Russian	1
Syrian refugee	2
